# Rumination and “hot” executive function of middle school students during the COVID-19 pandemic: A moderated mediation model of depression and mindfulness

**DOI:** 10.3389/fpsyt.2022.989904

**Published:** 2022-11-14

**Authors:** Ying Li, Guiping Qu, Huiyan Kong, Xiaobo Ma, Lei Cao, Tiantian Li, Yue Wang

**Affiliations:** School of Education, Zhengzhou University, Zhengzhou, China

**Keywords:** COVID-19, rumination, depression, mindfulness, executive function

## Abstract

**Background:**

The outbreak of COVID-19 had a widely negative effect on adolescents’ academics, stress, and mental health. At a critical period of cortical development, adolescents’ cognition levels are highly developed, while the ability of emotion control is not developed at the same pace. Faced with negative emotions such as stress and social loneliness caused by COVID-19, adolescents’ “hot” executive function encounters severer emotional regulation challenges than ever before.

**Objective:**

The present study established a moderated mediation model to investigate the impact of rumination on “hot” execution function among Chinese middle school students during the COVID-19 pandemic, and the specific role of depression and mindfulness in the association.

**Materials and methods:**

This cross-sectional study was conducted on 650 students recruited from a province in central China. The participants completed questionnaires and experiment between July 2021 and August 2021. Rumination Responses Scales, Self-rating Depression Scale, and Mindful Attention Awareness Scale were used to measure the level of rumination, depression, and mindfulness. The reaction time and accuracy of the emotional conflict experiment were recorded to reflect the “hot” executive function.

**Results:**

The results of the moderated mediation model indicated that rumination of middle school students significantly and positively predicted depression in adolescents (β = 0.26, *p* < 0.001). Meanwhile, the indirect effect of depression on the relationship between rumination and “hot” executive function was significant; depression partially mediated this relationship (word-face congruent condition: β = −0.09, *p* < 0.01; word-face incongruent condition: β = −0.07, *p* < 0.05). Furthermore, mindfulness buffered the association between rumination and depression, according to moderated mediation analysis (β = −0.11, *p* < 0.001). For adolescents with low levels of mindfulness, the relationship was substantially stronger.

**Conclusion:**

In the context of the COVID-19 pandemic, middle school students’ rumination would lead to depression, which can negatively impact their “hot” executive function. Besides, mindfulness could resist the adverse effect of rumination on depression. The educators should pay more attention to students’ mental health, provide targeted strategies that boost mindfulness to promote their cognitive flexibility, and thus protect the normal development of their executive function during crisis events.

## Introduction

For nearly 3 years, COVID-19 has been sweeping the globe, dramatically changing everyone’s life. Due to the community quarantine and the “Online Learning at Home” education policies, middle school students are one of the social groups most affected by the epidemic. Relative to adults, adolescents exhibit stronger “bottom-up” affective reactivity in response to socially relevant stimuli (1). Meanwhile, it is a sensitive and window period of maturation of brain functions, especially the development of the executive function. As a general control system, the executive function (EF) regulates and controls adolescents’ cognitive processes, and further determines their cognitive and social function. Although EF is booming during this time, it is still affected by adolescents’ external environment and themselves. Therefore, it is urgent and necessary to profoundly investigate the influencing factors and underlying mechanism of adolescents’ EF under the worldwide spread of the pandemic.

### Rumination and “hot” executive function

Executive function is an umbrella term that refers to higher-order cognitive processes and behavioral competencies, including planning, cognitive flexibility, social cognition [e.g., empathy and theory of mind (ToM)], and emotion regulation (2). According to the neurodevelopmental model of EF, it can be divided into “cold” and “hot” functions. The former is an unemotional cognitive process related to the activation of the dorsolateral prefrontal cortex, while the latter contains an affective or reward system involving the recruitment of the orbitofrontal cortex (OFC) (3). Previous studies have shown that when compared to cold EF which focuses on purely non-contextualized and non-emotional cognitive activities, hot EF is mainly based on emotional involvement, which requires individuals to assess the emotional meaning of stimuli flexibly and is used to deal with problems related to motivation and emotion regulation, including both emotional decision-making and emotional conflict (4–6). Specifically, hot EF is a goal-directed, future-oriented cognitive process elicited in contexts that engender emotion, motivation, and tension between immediate gratification and long-term rewards (7).

Rumination is engaged in a passive focus on one’s symptoms of distress and on the possible causes and consequences of these symptoms (8). Recent studies have revealed that adolescents will perform worse on cognitive tasks with repetitive and recurrent negative thinking about their personal concerns and upsetting experiences (9), indicating the negative effect of rumination on EF (10). According to the Resource allocation theory, negative thoughts of rumination deplete limited cognitive abilities, which would otherwise be directed toward task-relevant processes (11–13). This view is confirmed in the studies that when negative information is maintained in working memory, it is difficult for participants to exert executive control over it. This is due to the limitations of cognitive resources; on one hand, suppressing negative stimuli takes up resources that had been used to process the stimuli. On the other hand, the entrance of the stimulus into working memory lets the adolescent pay greater attention to it. This may account for the continual perpetuation of the rumination cycle (14, 15). In addition, the attentional scope model of rumination holds that rumination can lead to difficulty in working memory updating. To be specific, adolescents with a high level of rumination may have difficulty in processing and disengaging from negative information (16–18).

Although a large number of studies demonstrated the link between rumination and EF, previous studies mainly focused on the “cool” EF, yet the effect of rumination on hot EF has not received much attention (19, 20). Several recent studies have shown that people engaging in higher levels of rumination may lead to impulsive behaviors such as binge eating or drinking (21), and non-suicidal self-injury (22), indicating the impairment of executive control abilities by rumination. It was also found that adolescents with high rumination exhibited deficits in the inhibition of negative emotional stimuli when completing the Affective Go/No-go task (23). The task examines the planning and inhibitory control components of individuals’ EF with the necessary requirement of highly emotional involvement. As mentioned above, the difference between cold and hot EF is that hot EF prefers to address emotion-related information rather than abstract cognitive information. These findings further indicate the inner link between rumination and hot EF. Specifically, adolescents’ ability to accurately process emotional information will be inhibited when they repeatedly and passively think about their negative emotional condition, the causes, and the consequences of their negative emotions. Based on the theoretical and literature review, we proposed Hypothesis 1: rumination can negatively predict the hot EF of adolescents.

### Depression as a mediator

If rumination can negatively affect the hot EF in adolescents, then the question we urgently need to address is: what is the underlying psychological mechanism for this effect? Previous research has focused on how rumination interferes with the rational allocation of limited attentional resources and prevents individuals from selectively attending to the information in the current task. However, the hot EF emphasizes the processing of motivational and emotional information. Thus, rumination not only affects emotional processing by interfering with attention, but more likely prevents the processing of emotional tasks by directly bringing about disruptive emotions. In fact, a large number of studies have shown a close relationship between rumination and depressed mood. Individuals who fall into rumination tend to focus their attention on depressive symptoms and on behaviors or thoughts related to depressed mood. According to Beck’s influential cognitive theory (24), individuals with depressive symptoms also have similar negative schema, manifested as the mental representations of past negative events and negative evaluations of the self for a long time (25, 26). Moreover, the response style theory of depression proposes that rumination, as a trait-like response style to distress, will amplify and prolongs individuals’ existing negative emotional state and associated negative thinking, ultimately leading to depression (27).

Empirical studies have also proved the relationship between rumination and depression in different dimensions. For example, a longitudinal study revealed that high rumination positively predicted the development, maintenance, and recurrence of depression (28, 29). Neuroimaging studies further provide physiological evidence for a correlation between rumination and depression, showing that the activation of the subgenual cingulate area, which has been proved to be associated with rumination in depression patients, was significantly stronger than that in healthy controls (30). A cross-sectional study with adolescents also showed that increased levels of stress and rumination in early adolescence predicted depression (31). Adolescents with high levels of rumination always get caught up in their current distress and engage in repetitive thoughts about negative events, causing them unable to find positive strategies to fix the problem, which leads to a more intensely depressed mood (32, 33). During COVID-19, when the epidemic kept people from fulfilling purposes, adolescents who constantly thought about why they failed would slip into rumination and increase the risk of depression. That is to say, the negative events associated with the epidemic may lead to negative beliefs in adolescents, which would significantly increase the risk of adolescent depression.

Besides the relationship between rumination and depression, some studies have also focused on the impact of depression on the EF, which obtains ambiguous results. For example, researchers found that when assessing the ability to sustain attention in children and adolescents with depressive disorders using the continuous performance test (CPT), the patients made more errors and responded more slowly than participants under healthy control (34). Indeed, it has been found that error rates of positive stimuli were higher for depression groups than healthy controls, while the performance of the negative stimulus showed no difference between the two. Besides, depressed participants appeared to respond to negative stimuli more quickly than healthy ones (35, 36). Moreover, depressed patients had significantly longer reaction times to negative mood backgrounds than to neutral backgrounds in the Emotional n-back task (37). Neuroimaging evidence also confirms the negative impact of depression on EF: there are functional abnormalities in key brain regions responsible for EF in depressed adolescents (38). However, the results of other studies have shown little impairments in some sub-dimensions of the EF for depressive patients such as response inhibition, selective attention, and verbal WM (working memory) (39–41). The reason for the inconsistent results may be that different research did not distinguish between the hot and cold components of the EF. Since depression has been associated with an attention bias toward negative stimuli (42), it may be more associated with a greater sensitivity to the processing of emotional information than to non-emotional cognitive activities. Therefore, we argue that depression may play a particular role in the connection between rumination and adolescents’ hot EF, specifically, whether rumination affects hot EF through the mediating role of depression (Hypothesis 2).

### Mindfulness as a moderator

To fully explore the underlying mechanisms that promote adolescents’ mental health and cognitive development, it is not enough to only analyze the risk factors but is equally important to look for protective factors from external communities and adolescents themselves. Changes in the surrounding environment caused by the epidemic bring floods of negative emotions to adolescents, which may lead them to rumination. More importantly, there is an unprecedented challenge to adolescents’ emotion management as well as EF development. However, not all adolescents facing these conditions will go to the depths of depression, causing irreversible damage to cognitive development. More and more evidence from clinical and empirical studies has proved the inherent positive relationship between mindfulness and adolescent mental health. Mindfulness, acting as a positive factor, entails directing attention to the present moment in a non-judgmental and accepting way, which is contrary to rumination solely focused on and guided by past negative experiences. Hence, mindfulness may mitigate the negative effects of rumination on adolescent emotion and cognitive control ability. First, substantial empirical research has supported that enhancing mindfulness can decrease rumination and reduce depressive symptoms in individuals. For instance, completers of the MBSR (mindfulness-based stress reduction) class showed increases in mindfulness and overall wellbeing as well as decreases in rumination and symptoms of depression (43). By shifting their attention to the present moment from rumination, mindfulness-based interventions can reduce the possibility of depression relapse by producing a significant or moderate reduction of rumination (44). Second, mindfulness also creates cognitive diffusion (45), which helps individuals to realize that they are in a dysfunctional thinking pattern, such as rumination.

According to the information-processing model, mindfulness can also enhance the capacity to regulate emotion, which is a core element of hot EF (46). In addition, the reawakening model indicates that mindfulness can help individuals improve their cognitive flexibility by expanding attentional space and changing their maladaptive thinking patterns, which ultimately reduces the incidence of depression (47). Besides, mindfulness is conceptualized in terms of self-regulation of attention (48), which can be fully applied to adjust individuals’ negative emotions and thus shape positive perceptual experiences. Researchers using rs-fMRI and local synchronization measurements found that major dispositional mindfulness correlated to left OFC, while local synchronization levels of the OFC are widely assumed to predict activation of positive emotions in the brain (49). Therefore, we predict that mindfulness would moderate the association among rumination, depression, and hot EF (Hypothesis 3).

### The present study

Based on previous studies and the theoretical framework, we argue that the association of rumination with adolescents’ hot EF can be mediated by depression. Firstly, the impact of COVID-19 has significantly changed the learning and living environment of Chinese adolescents. Specifically, when they are at home without face-to-face contact with teachers and friends, their feelings of isolation increase (50). However, when they return to school, the strict epidemic prevention policy leads to the reduction of their leisure time and the increase of their study time, which increases their academic stress. In conclusion, based on previous studies, it was found that the rumination and depression of Chinese adolescents increased significantly during the epidemic (51, 52). Therefore, in this study, we explore these two variables hoping to find their underlying cognitive mechanisms and promptly intervene on them.

Secondly, adolescence is a critical window for adolescents’ cognitive development, and EF, as a general control system, has a fundamental role in the development of adolescents’ cognitive and social function (2). As a two-dimensional concept, on the one hand, EFs include non-emotional cognitive functions such as inhibition control, memory refreshment, and cognitive flexibility; on the other hand, adolescents also rely on EFs to make their judgments on emotional stimuli and make emotional decisions. The latter has been insufficiently explored in previous studies, so this study hopes to add and expand on this area (4, 6, 7).

Finally, the mechanisms by which rumination affects hot EF are unclear, thus the present study includes depression as a mediating variable. At the same time, the level of mindfulness in adolescents was considered a protective factor to mitigate the effects of both types of negative emotions on hot EF. The reason for this is that the mechanisms of rumination and depression have something in common. Moreover, neurophysiology has found supporting evidence that the brain regions involved in both overlaps. Moreover, mindfulness, as a positive psychological variable, has also been suggested to help shift adolescents’ attention away from negative events, which means that it increases the flexibility of emotional cognition. Therefore, we suggest that adolescents’ rumination and depression would have a significant negative impact on their hot EF, while their level of mindfulness could mitigate this negative effect.

**FIGURE 1:**
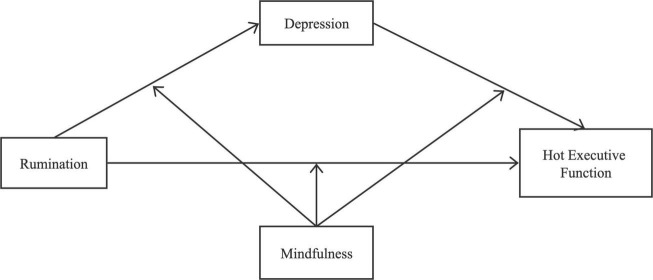
The proposed theoretical model.

Thus, in this study, we aimed to investigate both the association and the underlying mechanisms between rumination and adolescents’ hot EF, with a focus on the potential mediating effect of depression. We expected to find direct and negative relations between rumination and depression in the hot EF of adolescents. Finally, we also expected the negative relationship to be mediated by mindfulness (Figure 1). Based on the literature review, we propose a moderated mediation model.

## Materials and methods

### Participants

In this study, we recruited students from one randomly selected junior high school in a developed city in Henan province located in central of China. The Chinese education system is a 9-year compulsory education system in which children graduate from primary school and move on to middle school to complete the remaining 3 years of compulsory education. At the stage of middle school, the average age of adolescents is about 12–15 years old, and they are allowed to go home every 2 weeks due to the impact of the epidemic. A total of 650 questionnaires were collected through our survey, and 583 were valid, yielding a valid response rate of 89.69%. In addition, we also removed the extreme data with response times below 300 ms or over 1500 ms and invalid data with a total correct rate of less than 65% in the Word-Face Stroop experiment. In the end, 516 valid data were retained. These respondents consisted of 268 boys (51.9%) and 248 girls (48.1%), 261 (50.6%) were from grade seven, 138 (26.7%) were from grade eight, and 117 (22.7%) were from grade nine, only children accounted for 20.50%, and non-only children accounted for 79.50% (Table 1).

**TABLE 1:** Demographic characteristics of respondents.

		*N*	%
Gender	Male	268	51.90%
	Female	248	48.10%
Grade	Seventh-grade	261	50.60%
	Eighth-grade	138	26.70%
	Ninth-grade	117	22.70%
Family	Only-children	106	20.50%
	Non-only children	410	79.50%

All students took our survey voluntarily and they were told they could withdraw from the survey at any time. In addition, written informed consent was obtained from all participants prior to the start of the questionnaire and experiment. The study procedures were approved by the Zhengzhou University Ethics Committee. The study complied with the principles of the 1964 Declaration of Helsinki (including its subsequent amendments or similar ethical standards).

### Rumination responses scales

The Rumination Responses Scale (RRS) was used to assess levels of rumination. Han and Yang (53) interpreted a Chinese language version of the RRS, which has been widely employed in Chinese culture with satisfactory reliability and validity (53). This scale consists of 22 items with three dimensions, including symptom rumination, brooding, and reflective pondering. Participants were required to respond to each item on a 4-point Likert scale ranging from 1 (almost never) to 4 (almost always). This scale exhibited good reliability in the present study (Cronbach’s α = 0.93).

### Self-rating Depression Scale

Depression was measured by the Chinese version of Zung’s Self-Rating Depression Scale (SDS) (54). The SDS has 20 items that assess emotional, physiological, psychomotor, and psychological imbalances. Each item was measured by a 4-point Likert scale. The total scores ranged from 25 to 100 (20 × 1 × 1.25 to 20 × 4 × 1.25), the higher the score, the more severe the depressive symptoms. The Cronbach’s α coefficient of the SDS was 0.75 in this study.

### Mindful attention awareness scale

The Chinese version of the Mindfulness Attention Awareness Scale was revised by Chen et al. to measure mindfulness based on “current attention and awareness” (55). There are 15 items, each of which is scored from 1 to 6, with high scores indicating higher levels of awareness and attention. The Cronbach’s α coefficient for the MAAS in this study was 0.90, which was considered reasonable.

### The Word-Face Stroop paradigm

#### Stimuli

Compound stimuli consisted of pictures with facial expressions and affective words were used in this experiment. Picture stimuli were selected from CFAPS [the Chinese Face Affective Picture System; (56)] and were widely used in previous studies to investigate executive function of emotional conflict (57, 58). The valence and arousal of these expressions were evaluated on a 9-point scale by 24 middle school students who did not participate in the formal experiment. Twenty-four positive and twenty-four negative expressions were selected according to the rating result, with photos of each valence comprising 12 male and female faces, respectively. There were significant differences in the valence and arousal between positive and negative expressions (Valence: *M*_*positive*_ = 7.24, *M*_*negative*_ = 2.67, *F* = 1.45, *p* < 0.001; arousal: *M*_*positive*_ = 5.46, *M*_*negative*_ = 4.39, *F* = 4.56, *p* < 0.001). Affective words were selected from the word database of Yao’s study. After evaluating valence and arousal, 48 affective words (24 positive and 24 negative words) were selected as the final experimental materials from 60 original materials. There were significant differences in valence and arousal between positive and negative words (Valence: *M*_*positive*_ = 7.54, *M*_*negative*_ = 2.23, *F* = 9.40, *p* < 0.001; arousal: *M*_*positive*_ = 6.66, *M*_*negative*_ = 6.23, *F* = 2.76, *p* < 0.001).

Ninety-six compounded stimuli were prepared with negatively or positively valenced words in prominent red color superimposed on pictures. The word and facial expression of a compound stimulus were either congruent [e.g., “彩虹” (rainbow) was superimposed onto a positive expression] or incongruent [e.g., “诈骗” (swindle) was superimposed onto a positive expression]. The main experimental stimuli conclude consisted of 40 incongruent trials and 40 congruent trials (in each of the two blocks). Before the formal experiment, there were another 16 trials containing both experimental conditions as a practicing part.

#### Procedure

**FIGURE 2:**
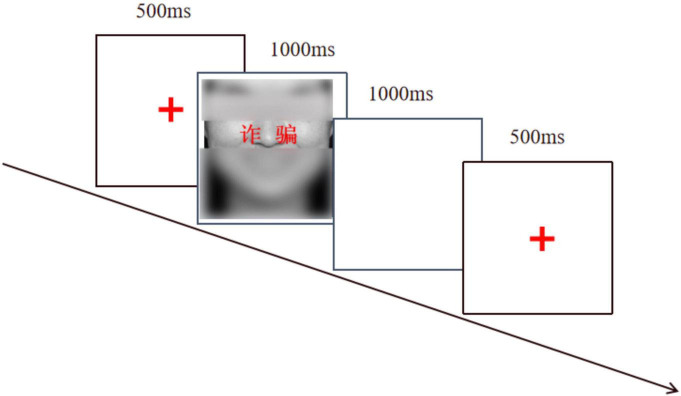
The experimental trial of the Word-Face Stroop task.

The experiment was programmed and presented using E-Prime 2.0 and was conducted within a multimedia classroom. The stimuli were shown on the gray background at the center of a laptop monitor, from which participants were seated 60 cm away. Each trial begins with a fixation “+” for 500 ms. Then, the face-word stimuli appeared in the center of the screen. Participants were asked to judge the valence of the words as quickly as possible, which necessitated inhibition of the emotion induced by the facial expression. Half of the participants were told to press the F key for the positive valence and to press the J key for the negative valence, while the other half were given a reversed response. Then a blank screen was presented for 1000 ms and followed by the next trial. If the participant does not respond after 2000 ms, the program will record it as an incorrect response (see Figure 2).

The administration process of this study was conducted by two graduate students in psychology as the main examiners. The test was conducted in a computer classroom and we received permission from students, teachers, and school officials before the administration. Students were asked to answer the questions independently according to their real feelings, and the test was collected on the spot after completion. After the questionnaires and the word-faces Stroop test were collected, descriptive statistics, independent samples *t*-test, one-way ANOVA, repeated measures ANOVA, and product-difference correlation analysis were conducted using SPSS 22.0, as well as mediating effects tests and moderated mediating effects tests using Model 59 and Model 7 of Hayes’ PROCESS macro program.

## Results

### Preliminary analyses

Table 2 shows means, SDs, and Pearson correlations for the study variables. As the results demonstrated, rumination was positively correlated with depression and negatively correlated with mindfulness and congruent/incongruent condition accuracy. In addition, depression negatively correlated with congruent/incongruent conditions accuracy. Notably, the reaction time of congruent/incongruent conditions was only negatively correlated with incongruent condition accuracy and has no significant correlations with rumination, depression, and mindfulness. Therefore, the following analysis used congruent/incongruent condition accuracy as the indicator for evaluating the hot EF of adolescents.

**TABLE 2:** Means, standard deviations, and correlations of the main variables.

	M	SD	1	2	3	4	5	6
(1) Rumination	41.72	12.22	–					
(2) Depression	48.52	9.28	0.54**	–				
(3) Mindfulness	64.59	14.33	−0.60**	−0.57**	–			
(4) ICA	0.85	0.12	−0.19**	−0.18**	0.14**	–		
(5) CCA	0.92	0.07	−0.22**	−0.22**	0.15**	0.53**	–	
(6) ICRT	748.11	148.9	−0.04	−0.02	−0.01	−0.17**	0.07	–
(7) CCRT	710.23	140.85	−0.04	−0.01	0.00	−0.17**	0.04	0.95**

*N* = 516; *p < 0.05; **p < 0.01.

CCA, congruent condition accuracy; ICA, incongruent condition accuracy; CCRT, congruent condition reaction time; ICRT, incongruent condition reaction time.

### Testing for mediation effect

The result showed that rumination was negatively correlated with CCA and ICA supporting Hypothesis 1 (CCA: β = −0.13, *t* = −5.00, *p* < 0.01, 95%CI = [−0.19, −0.07]; ICA: β = −0.13, *t* = −4.49, *p* < 0.001, 95%CI = [−0.19, −0.07]). In Hypothesis 2, we assumed that depression mediates the relationship between rumination and hot EF. The hypothesis was tested with Model 4 of the PROCESS macro. As Table 3 showed, rumination was positively associated with depression (β = 0.52, *t* = 14.43, *p* < 0.001, 95%CI = [0.45, 0.59]), which in turn was negatively related to hot EF (CCA: β = 0.08, *t* = −2.66, *p* < 0.01, 95%CI = [−0.14, −0.02]; ICA: β = −0.09, *t* = −2.92, *p* < 0.01, 95%CI = [−0.15, −0.03]). In the meantime, the negative direct association between rumination and hot EF remained significant. The result of the mediation effect analysis supported Hypothesis 2, verifying that depression partially mediated the relationship between rumination and hot EF.

**TABLE 3:** The mediation effect and moderated mediation effect of rumination on hot executive function (EF).

Predictors	Model 1 (Depression)		Model 2 (CCA)		Model 3 (ICA)	
	β (95%CI)	*t*	β (95%CI)	*t*	β (95%CI)	*t*
Rumination	0.26 (0.17, 0.34)	6.00***	−0.08 (−0.14, −0.02)	−2.66**	−0.09 (−0.16, −0.02)	−2.67**
Depression			−0.09 (−0.15, −0.03)	−2.92**	−0.07 (−0.14, −0.01)	−2.11*
Mindfulness	−0.38 (−0.47, −0.30)	−8.95***				
Rumination Mindfulness	−0.11 (−0.17, −0.05)	−3.62***				
*R* ^2^	0.400		0.06		0.05	
*F*	113.96***		16.87***		12.38***	

*N* = 516; *p < 0.05, ** *p* < 0.01, *** *p* < 0.001.

CCA, congruent condition accuracy; ICA, incongruent condition accuracy.

### Moderated mediation effect analysis

Model 59 of the SPSS Macro Program PROCESS (version 2.13) was used to test whether there was a moderating effect of mindfulness in the mediated model. Statistical results showed that only the interaction term between rumination and mindfulness had a significant negative predictive effect on depression in adolescents. The interaction term between rumination and mindfulness and the interaction term between depression and mindfulness did not have a significant effect on the hot EF.

**FIGURE 3:**
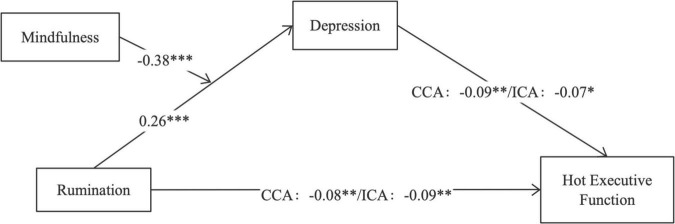
The final moderated mediation model. *p < 0.05, **p < 0.01, ***p < 0.001.

Further, Model 7 of the SPSS Macro Program PROCESS (version 2.13) was used to test the moderating effect of mindfulness. According to the method proposed, the relationship between rumination and hot EF, the mediating role of depression, and the moderating role of mindfulness are discussed (59). With depression as the dependent variable, rumination significantly predicted depression positively (β = 0.26, *t* = 6.00, *p* < 0.001), mindfulness significantly negatively predicted depression (β = −0.38, *t* = −8.95, *p* < 0.001), and the interaction between rumination and mindfulness significantly negatively predicted depression of middle school students (β = −0.11, *t* = −3.62, *p* < 0.001). This shows that mindfulness moderates half of the mediating role between rumination and depression. In the second equation, with the accuracy of congruent/incongruent condition as the dependent variable, depression significantly negatively predicted the accuracy of congruent/incongruent condition (β = −0.09, *t* = −2.92, *p* < 0.01; β = −0.07, *t* = −2.11, *p* < 0.05), rumination significantly negatively predicted the accuracy of congruent/incongruent condition (β = −0.08, *t* = −2.66, *p* < 0.01; β = −0.09, *t* = −2.67, *p* < 0.01). According to the results of moderated mediated effects, mindfulness regulates the first half path of the mediation process. Therefore, mindfulness plays a moderating role in the first half of the mediating effect, and Hypothesis 3 is verified. The specific model is shown in Figure 3.

**FIGURE 4:**
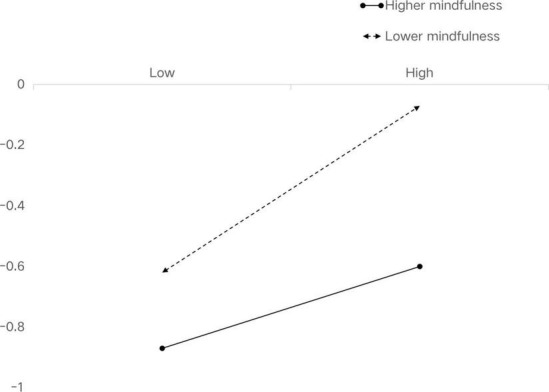
Association between rumination and depression at higher and lower levels of mindfulness.

The product (interaction term) of rumination and mindfulness had a significant predictive effect on depression (β = −0.11, *t* = −8.95, *p* < 0.001). The result supported Hypothesis 3. To further portray the interaction, we conducted simple slope plots and calculated beta coefficients at −1SD and +1SD from the mean of mindfulness (Figure 4). The result of simple slope tests showed that for middle school students with a lower level of mindfulness, the influence of rumination on depression had a steeper slope, meaning it was statistically significant (β_*simple*_ = 0.36, *t* = 2.68, *p* < 0.01). For middle school students with a higher level of mindfulness, the influence of rumination on depression was positively and statistically significant (β_*simple*_ = 0.15, *t* = 2.68, *p* < 0.01).

## Discussion

The present study investigated the relationship and the underlying mechanism between rumination and hot EF in Chinese middle school students during the spread of COVID-19. Our findings show that rumination can negatively predict adolescents’ hot EF with the mediating role of depression. Furthermore, mindfulness played a moderating role in the effect of rumination on depression.

### The mediating role of depression

Prior research has shown that both rumination and depression are crucial risk factors for adolescents’ EF (16, 60, 61). Nevertheless, on the one hand, these studies have developed independently of each other, neglecting the possible linkage between rumination and depression. Researchers followed 200 adolescents for 15 months and found that adolescents with higher levels of rumination showed reduced selective attention and attentional shifts at follow-up compared to baseline, without finding a predictive effect of depression levels (62). On the other hand, they explored almost solely the cold part of EF, lacking evidence from the hot EF. The present study highlights depression as a critical carrier of the impact of rumination on hot EF. Therefore, depression is not only an outcome of rumination but also causes damage to the hot EF of adolescents. These findings uncover why rumination may negatively predict the hot EF of adolescents.

Firstly, rumination directly affects adolescents’ hot EF, which manages emotions and repairs negative emotions. It has been suggested that cognitive inhibition is a key mechanism for repairing emotions, and to accomplish this process requires individuals to exert effective attentional control over negative emotions in working memory. Caught in repetitive negative thoughts, rumination impairs this function due to depleting cognitive resources and affecting the individual’s ability to solve emotional problems (63). According to the cognitive construct of the emotion regulation strategy, the effectiveness of emotion regulation depends on the working memory capacity to reevaluate current negative events, meaning to modify or update these negative thoughts with new neutral or positive information (20). When adolescents fall into rumination, their working memory is occupied with negative information (64), which hinders effective emotional regulation and then causes impairment to the hot EF. Moreover, the researchers measured the level of rumination in 52 adolescents before asking them to complete the Affective Go/No-go task and found that it was difficult that inhibit negative information when switching from negative to positive blocks on an Affective Go/No–go task (23).

Secondly, consistent with the negative cognitive model, the mediating pathway suggests that rumination positively predicts the level of depression in adolescents. According to the response style theory (64), when faced with stress or adverse life events, individuals exhibiting high levels of rumination usually regard the problems more negatively and are divorced from adaptive problem-solving behaviors that serve to address the source of the issue (65). Besides, adolescents with high levels of rumination always get caught up in their current distress and engage in repetitive thoughts about negative events, especially in the social epidemic environment. This cognitive style causes them to be unable to find positive strategies to fix the problem, which eventually increases the risk of depression occurrence (66). In addition, the study revealed that depression plays a special role between rumination and the hot EF of adolescents. For adolescents who are at a critical stage of mental development, depression will destroy their ability to regulate their emotions and cause them to make a series of decisions that are detrimental to their future development (67). According to emotion regulation theory, effective emotion regulation requires an additional cost in cognitive resources (68). When rumination keeps adolescents focusing on their depressive symptoms (21), it may imply an increase not only in self-understanding but also in negative cognition. Therefore, this will occupy adolescents’ cognitive resources for effective problem solving and will correspondingly impair their hot EF (69).

### The moderating role of mindfulness

Although rumination of adolescents may be significantly associated with hot EF through the mediating role of depression, this relationship is not stable and unchanging. Therefore, the present study meanwhile explored potential moderating variables that may influence the relationship among rumination, depression, and hot EF. By exploring the positive factors, we can implement effective interventions for negative thinking in adolescents to improve their ability to cope with frustrating events and maintain their mental health. Consistent with our hypothesis, mindfulness moderated the relationship between rumination and depression. Specifically, the higher the level of mindfulness in adolescents, the weaker the prediction from rumination to depression. Previous research has also found that high personality mindfulness can break down the maintenance of rumination and can reduce the risk of depression relapse (70). As we proved above, focusing on negative information may lead to continuous negative emotions like depression and even impair the cognitive ability to deal with emotion-related tasks. However, a high level of mindfulness helps adolescents to consciously bring their attention back to the internal and external experiences occurring in the present moment and thus move away from the cognitive control of negative emotions, thereby lowering the adverse effects of negative emotions on hot EF. This finding is consistent with previous research which also verified that mindfulness training could not only improve its own level but, more importantly, reduce the level of rumination (43).

The result that mindfulness as a protective factor weakens the adverse effects of rumination on depression in adolescents can be explained from several aspects. Firstly, COVID-19 causes mass negative emotions in adolescents because they are at a unique stage of physical and mental development. Therefore, the contradiction and conflict between the internal and external environment make them fall into rumination, which triggers depression (29, 71). However, according to cognitive flexibility theory, mindfulness advocates the conscious awareness of the present moment and non-judgmental acceptance, which is different from the rumination cognitive style that focuses on negative information and bad situations (16, 61). Adolescents with higher levels of mindfulness can more quickly be aware of changes in their surroundings and take flexible responses to get themselves out of negative emotions as soon as possible. Secondly, positive psychological approaches treat mindfulness as one of the positive variables that enable individuals to increase their attentional flexibility, cognitive abilities, and a variety of positive psychological resources such as mental toughness and self-esteem (72). Using a wealth of psychological resources, adolescents can counteract the effects of the external environment on their emotions. This means that mindfulness can motivate adolescents to become aware of their surroundings in an objective, non-judgmental, and accepting manner, thus breaking the rumination and alleviating their depression (73). When uncontrollable events occur, mindfulness can be used to adjust teens’ cognition to buffer the adverse consequences caused by the crisis event.

### Strength and limitations

Firstly, adolescent rumination and EF are two areas of research with rich empirical findings, while studies focusing on the effects of rumination on hot EF in adolescents are scarce, especially in the uncertain situation of a worldwide pandemic. This study explored this using a combination of measurement and experimental research methods. As hot EF is a complex high-level cognitive process under consciousness, it is difficult to measure through self-report scales and is more accurately reflected through performance on real-time cognitive processing tasks. The present study explored it using the Word-Face Stroop paradigm, which improved the accuracy of the study. More importantly, the findings of this study show that adolescents’ rumination can directly affect individuals’ hot EF, as well as indirectly by impacting depression levels. Adolescent individuals are maturing physiologically at a rapid pace and going to become true adults, which often endures various internal and external conflicts and contradictions psychologically. This is the objective reason why they are prone to rumination. They tend to escape whenever they encounter insurmountable obstacles, and the feeling of powerlessness also induces the possibility of depression.

More critically, adolescence is a window of opportunity for intervention and improvement of the hot EF, and the findings of this study confirm that at this stage, the development of hot EF must be given attention by all educational authorities. First of all, educators need to grasp the psychological developmental characteristics of junior high school students. In regular school mental health education, their attention should be paid to preventing students from developing negative cognitive patterns of rumination. For example, they can increase their positive emotional experiences through some recreational activities, and they can also provide interview exchanges and psychological counseling to help them learn ways to reasonably dissipate negative emotions. In addition, when young people experience unpleasant events, educators should guide them to resolve the issue through adaptive means, such as transference or seeking solutions, rather than repeatedly dwelling on what caused the event to occur. By preventing the occurrence of rumination, the adolescent’s hot EF are protected from developing properly.

In addition to school educators, we expect the families of teenagers to give the necessary attention to their mental health. Parents should pay attention to the psychological needs behind their behavioral problems, and timely relieve and diffuse the confusion that cannot be resolved due to the physical and mental limitations of their teens. At the same time, parents of adolescents should provide them with a peaceful and love-filled growing family, respect their ideas and foster effective methods of communication, thus enhancing the positive psychological resources of these minors.

In addition to the two perspectives above, we should also note another important finding of this study, which is that mindfulness plays a moderating role among rumination, depression, and hot EF. For individuals with high levels of mindfulness, the predictive effect of rumination on depression was diminished. This is an important insight into the need for targeted measures to improve the level of mindfulness in junior high school students. In detail, a mindfulness program is necessary in order to guide students to internalize and apply mindfulness in their daily lives, thereby extending the cognitive flexibility of individuals, guiding them to remain aware and alert to the present moment and to be able to allocate more of their limited cognitive resources to positive aspects. This will motivate young people to adopt positive coping strategies to detach themselves from undesirable cognitive patterns or negative emotions as soon as possible, contributing to their physical and mental health and hot EF.

Despite revealing meaningful insights, the present study still has some limitations that need to be noted. Firstly, this study is a cross-sectional study, and given the current broader epidemiological context, a cross-sectional survey is far from adequate to capture the psychological state of adolescents. COVID-19 has in fact affected our participants for up to 3 years, and this long-term change in their life circumstances will have a lifelong impact on the development of their mindset and EF. Therefore, a longitudinal study or a cross-lagged approach to confirm the impact of rumination on their EF would be a very important complement to the results of the cross-sectional study. Secondly, due to the impact of the prevention and control policy, only a small administrative region in central China was chosen as the source of participants, and the homogeneity of the source of participants hinders the extension of the findings to a more general group of adolescents. Therefore, in future studies, we hope that researchers will be able to compare the psychological states of adolescents across regions and cultures to increase the generalizability of the findings. Finally, we only measured adolescents’ levels of mindfulness, but lacked interventions to treat their levels of mindfulness. Therefore, this study is a *post-hoc* test and can only anticipate that increasing adolescents’ levels of mindfulness will improve their development of hot EF. Future studies can further validate the positive effects of mindfulness by comparing the mental states of the participants before and after the mindfulness intervention.

## Conclusion

In conclusion, this study presented the role of rumination and mindfulness in hot EF of middle school students. Rumination negatively affects adolescents’ hot EF by aggravating their depression, and mindfulness can moderate the effects of rumination on depression. Our results highlight that rumination of adolescents was dangerous during the COVID-19 pandemic, as it had a strong relationship with depression. Although rumination, as negative cognition, could increase depression, mindfulness could decrease depression.

## Data availability statement

The raw data supporting the conclusions of this article will be made available by the authors, without undue reservation.

## Ethics statement

The study procedures were approved by the Zhengzhou University Ethics Committee. Written informed consent was obtained from all participants prior to the start of the questionnaire and experiment.

## Author contributions

YL and YW contributed to conception and design of the study. HK organized the database. XM performed the statistical analysis. GQ and YL wrote the first draft of the manuscript. XM and GQ wrote sections of the manuscript. LC and TL read and contributed to manuscript revision. All authors contributed to the article and approved the submitted version.
